# Highlighting Entanglement of Cultures via Ranking of Multilingual Wikipedia Articles

**DOI:** 10.1371/journal.pone.0074554

**Published:** 2013-10-03

**Authors:** Young-Ho Eom, Dima L. Shepelyansky

**Affiliations:** Laboratoire de Physique Théorique du CNRS, IRSAMC, Université de Toulouse, UPS, Toulouse, France; University of Maribor, Slovenia

## Abstract

How different cultures evaluate a person? Is an important person in one culture is also important in the other culture? We address these questions via ranking of multilingual Wikipedia articles. With three ranking algorithms based on network structure of Wikipedia, we assign ranking to all articles in 9 multilingual editions of Wikipedia and investigate general ranking structure of PageRank, CheiRank and 2DRank. In particular, we focus on articles related to persons, identify top 30 persons for each rank among different editions and analyze distinctions of their distributions over activity fields such as politics, art, science, religion, sport for each edition. We find that local heroes are dominant but also global heroes exist and create an effective network representing entanglement of cultures. The Google matrix analysis of network of cultures shows signs of the Zipf law distribution. This approach allows to examine diversity and shared characteristics of knowledge organization between cultures. The developed computational, data driven approach highlights cultural interconnections in a new perspective.

Dated: June 26, 2013

## Introduction

Wikipedia, the online collaborative encyclopedia, is an amazing example of human collaboration for knowledge description, characterization and creation. Like the Library of Babel, described by Jorge Luis Borges [1], Wikipedia goes to accumulate the whole human knowledge. Since every behavioral ‘footprint’ (log) is recorded and open to anyone, Wikipedia provides great opportunity to study various types of social aspects such as opinion consensus [2], [3], language complexity [4], and collaboration structure [5]–[7]. A remarkable feature of Wikipedia is its existence in various language editions. In a first approximation we can attribute each language to an independent culture, leaving for future refinements of cultures inside one language. Although Wikipedia has a neutral point of view policy, cultural bias or reflected cultural diversity is inevitable since knowledge and knowledge description are also affected by culture like other human behaviors [8]–[11]. Thus the cultural bias of contents [12] becomes an important issue. Similarity features between various Wikipedia editions has been discussed at [13]. However, the cross-cultural difference between Wikipedia editions can be also a valuable opportunity for a cross-cultural empirical study with quantitative approach. Recent steps in this direction, done for biographical networks of Wikipedia, have been reported in [14].

Here we address the question of how importance (ranking) of an article in Wikipedia depends on cultural diversity. In particular, we consider articles about persons. For instance, is an important person in English Wikipedia is also important in Korean Wikipedia? How about French? Since Wikipedia is the product of collective intelligence, the ranking of articles about persons is a collective evaluation of the persons by Wikipedia users. For the ranking of Wikipedia articles we use PageRank algorithm of Brin and Page [15], CheiRank and 2Drank algorithms used in [16]–[18], which allow to characterize the information flows with incoming and outgoing links. We also analyze the distribution of top ranked persons over main human activities attributed to politics, science, art, religion, sport, etc (all others), extending the approach developed in [17], [19] to multiple cultures (languages). The comparison of different cultures shows that they have distinct dominance of these activities.

We attribute belongings of top ranked persons at each Wikipedia language to different cultures (native languages) and in this way construct the network of cultures. The Google matrix analysis of this network allows us to find interconnections and entanglement of cultures. We believe that our computational and statistical analysis of large-scale Wikipedia networks, combined with comparative distinctions of different languages, generates novel insights on cultural diversity.

## Methods

We consider Wikipedia as a network of articles. Each article corresponds to a node of the network and hyperlinks between articles correspond to links of the network. For a given network, we can define adjacency matrix 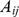. If there is a link (one or more quotations) from node (article) 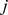 to node (article) 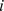 then 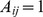, otherwise, 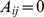. The out-degree 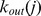 is the number of links from node 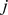 to other nodes and the in-degree 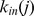 is the number of links to node 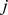 from other nodes.

### Google matrix

The matrix 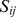 of Markov chain transitions is constructed from adjacency matrix 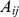 by normalizing sum of elements of each column to unity (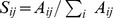, 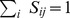) and replacing columns with only zero elements ( *dangling nodes*) by 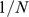, with 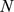 being the matrix size. Then the Google matrix of this directed network has the form [15], [20]:
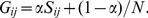
(1)In the WWW context the damping parameter 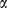 describes the probability 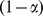 to jump to any article (node) for a random walker. The matrix 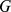 belongs to the class of Perron-Frobenius operators, it naturally appears in dynamical systems [21]. The right eigenvector at 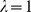, which is called the PageRank, has real non-negative elements 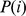 and gives a probability 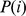 to find a random walker at site 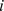. It is possible to rank all nodes in a decreasing order of PageRank probability 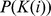 so that the PageRank index 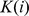 sorts all 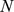 nodes 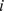 according their ranks. For large size networks the PageRank vector and several other eigenvectors can be numerically obtained using the powerful Arnoldi algorithm as described in [22]. The PageRank vector can be also obtained by a simple iteration method [20]. Here, we use here the standard value of 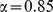 [20].

To rank articles of Wikipedia, we use three ranking algorithms based on network structure of Wikipedia articles. Detail description of these algorithms and their use for English Wikipedia articles are given in [17]–[19], [22].

### PageRank algorithm

PageRank algorithm is originally introduced for Google web search engine to rank web pages of the World Wide Web (WWW) [15]. Currently PageRank is widely used to rank nodes of network systems including scientific papers [23], social network services [24] and even biological systems [25]. Here we briefly outline the iteration method of PageRank computation. The PageRank vector 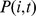 of a node 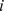 at iteration 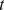 in a network of 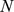 nodes is given by
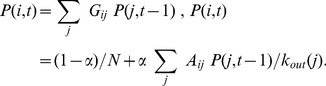
(2)


The stationary state 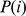 of 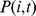 is the PageRank of node 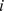. More detail information about PageRank algorithm is described in [20]. Ordering all nodes by their decreasing probability 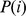 we obtain the PageRank index 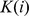.

The essential idea of PageRank algorithm is to use a directed link as a weighted ‘recommendation’. Like in academic citation network, more cited nodes are considered to be more important. In addition, recommendations by highly ranked articles are more important. Therefore high PageRank nodes in the network have many incoming links from other nodes or incoming links from high PageRank nodes.

### CheiRank algorithm

While the PageRank algorithm uses information of incoming links to node 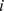, CheiRank algorithm considers information of outgoing links from node 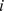 [16]–[18]. Thus CheiRank is complementary to PageRank in order to rank nodes in directed networks. The CheiRank vector 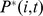 of a node at iteration time 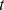 is given by
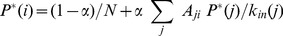
(3)We also point out that the CheiRank is the right eigenvector with maximal eigenvalue 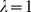 satisfying the equation 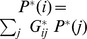, where the Google matrix 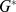 is built for the network with inverted directions of links via the standard definition of 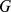 given above.

Like for PageRank, we consider the stationary state 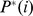 of 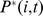 as the CheiRank probability of node 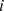 at 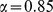. High CheiRank nodes in the network have a large out-degree. Ordering all nodes by their decreasing probability 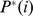 we obtain the CheiRank index 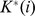.

We note that PageRank and CheiRank naturally appear in the world trade network corresponding to import and export in a commercial exchange between countries [26].

The correlation between PageRank and CheiRank vectors can be characterized by the correlator 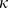 [16]–[18] defined by
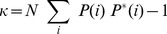
(4)The value of correlator for each Wikipedia edition is represented in Table 1. All correlators are positive and distributed in the interval 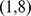.

**Table 1:** Considered Wikipedia networks from language editions: English (EN), French (FR), German (DE), Italian (IT), Spanish (ES), Dutch (NL), Russian (RU), Hungarian (HU), Korean (KO).

Edition	*N_A_*	*N_L_*	*κ*	Date
EN	3920628	92878869	3.905562	Mar. 2012
FR	1224791	30717338	3.411864	Feb. 2012
DE	1396293	32932343	3.342059	Mar. 2012
IT	917626	22715046	7.953106	Mar. 2012
ES	873149	20410260	3.443931	Feb. 2012
NL	1034912	14642629	7.801457	Feb. 2012
RU	830898	17737815	2.881896	Feb. 2012
HU	217520	5067189	2.638393	Feb. 2012
KO	323461	4209691	1.084982	Feb. 2012

Here 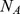 is number of articles, 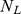 is number of hyperlinks between articles, 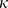 is the correlator between PageRank and CheiRank. Date represents the time in which data are collected.

### 2DRank algorithm

With PageRank 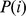 and CheiRank 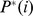 probabilities, we can assign PageRank ranking 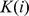 and CheiRank ranking 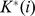 to each article, respectively. From these two ranks, we can construct 2-dimensional plane of 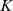 and 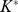. The two dimensional ranking 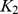 is defined by counting nodes in order of their appearance on ribs of squares in 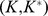 plane with the square size growing from 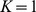 to 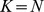 [17]. A direct detailed illustration and description of this algorithm is given in [17]. Briefly, nodes with high PageRank and CheiRank both get high 2DRank ranking.

## Data Description

We consider 9 editions of Wikipedia including English (EN), French (FR), German (DE), Italian (IT), Spanish (ES), Dutch (NL), Russian (RU), Hungarian (HU) and Korean (KO). Since Wikipedia has various language editions and language is a most fundamental part of culture, the cross-edition study of Wikipedia can give us insight on cultural diversity. The overview summary of parameters of each Wikipedia is represented in Table 1.

The corresponding networks of these 9 editions are collected and kindly provided to us by S.Vigna from LAW, Univ. of Milano. The first 7 editions in the above list represent mostly spoken European languages (except Polish). Hungarian and Korean are additional editions representing languages of not very large population on European and Asian scales respectively. They allow us to see interactions not only between large cultures but also to see links on a small scale. The KO and RU editions allow us to compare views from European and Asian continents. We also note that in part these 9 editions reflect the languages present in the EC NADINE collaboration.

We understand that the present selection of Wikipedia editions does represent a complete view of all 250 languages present at Wikipedia. However, we think that this selection allows us to perform the quantitative statistical analysis of interactions between cultures making a first step in this direction.

To analyze these interactions we select the fist top 30 persons (or articles about persons) appearing in the top ranking list of each of 9 editions for 3 ranking algorithms of PageRank, CheiRank and 2DRank. We select these 30 persons manually analyzing each list. We attribute each of 30 persons to one of 6 fields of human activity: politics, science, art, religion, sport, and etc (here “etc” includes all other activities). In addition we attribute each person to one of 9 selected languages or cultures. We place persons belonging to other languages inside the additional culture WR (world) (e.g. Plato). Usually a belonging of a person to activity field and language is taken from the English Wikipedia article about this person. If there is no such English Wikipedia article then we use an article of a Wikipedia edition language which is native for such a person. Usually there is no ambiguity in the distribution over activities and languages. Thus Christopher Columbus is attributed to IT culture and activity field etc, since English Wikipedia describes him as “italian explorer, navigator, and colonizer”. By our definition politics includes politicians (e.g. Barak Obama), emperors (e.g. Julius Caesar), kings (e.g. Charlemagne). Arts includes writers (e.g. William Shakespeare), singers (e.g. Frank Sinatra), painters (Leonardo da Vinci), architects, artists, film makers (e.g. Steven Spielberg). Science includes physicists, philosophers (e.g. Plato), biologists, mathematicians and others. Religion includes such persons as Jesus, Pope John Paul II. Sport includes sportsmen (e.g. Roger Federer). All other activities are placed in activity etc (e.g. Christopher Columbus, Yuri Gagarin). Each person belongs only to one language and one activity field. There are only a few cases which can be questioned, e.g. Charles V, Holy Roman Emperor who is attributed to ES language since from early long times he was the king of Spain. All listings of person distributions over the above categories are presented at the web page given at Supporting Information (SI) file and in 27 tables given in File S1.

Unfortunately, we were obliged to construct these distributions manually following each person individually at the Wikipedia ranking listings. Due to that we restricted our analysis only to top 30 persons. We think that this number is sufficiently large so that the statistical fluctuations do not generate significant changes. Indeed, we find that our EN distribution over field activities is close to the one obtained for 100 top persons of English Wikipedia dated by Aug 2009 [17].

To perform additional tests we use the database of about 250000 person names in English, Italian and Dutch from the research work [14] provided to us by P.Aragón and A.Kaltenbrunner. Using this database we were able to use computerized (automatic) selection of top 100 persons from the ranking lists and to compare their distributions over activities and languages with our case of 30 persons. The comparison is presented in figures S1,S2,S3 in File S1. For these 3 cultures we find that our top 30 persons data are statistically stable even if the fluctuations are larger for CheiRank lists. This is in an agreement with the fact that the CheiRank probabilities. related to the outgoing links, are more fluctuating (see discussion at [19]).

Of course, it would be interesting to extend the computerized analysis of personalities to a larger number of top persons and larger number of languages. However, the database of persons in various languages still should be cleaned and checked and also attribution of persons to various activities and languages still requires a significant amount of work. Due to that we present here our analysis only for 30 top persons. But we note that by itself it represents an interesting case study since here we have the most important persons for each ranking. May be the top 1000 persons would be statistically more stable but clearly a person at position 30 is more important than a one at position 1000. Thus we think that the top 30 persons already give an interesting information on links and interactions between cultures. This information can be used in future more extended studies of a larger number of persons and languages.

Finally we note that the language is the primary element of culture even if, of course, culture is not reduced only to language. In this analysis we use in a first approximation an equivalence between language and culture leaving for future studies the refinement of this link which is of course much more complex. In this approximation we consider that a person like Mahatma Gandhi belongs to EN culture since English is the official language of India. A more advanced study should take into account Hindi Wikipedia edition and attribute this person to this edition. Definitely our statistical study is only a first step in Wikipedia based statistical analysis of network of cultures and their interactions.

We note that any person from our top 30 ranking belongs only to one activity field and one culture. We also define local heros as those who in a given language edition are attributed to this language, and non-local heros as those who belong in a given edition to other languages. We use category WR (world) where we place persons who do not belong to any of our 9 languages (e.g. Pope John Paul II belongs to WR since his native language is Polish).

## Results

We investigate ranking structure of articles and identify global properties of PageRank and CheiRank vectors. The detailed analysis is done for top 30 persons obtained from the global list of ranked articles for each of 9 languages. The distinctions and common characteristics of cultures are analyzed by attributing top 30 persons in each language to human activities listed above and to their native language.

### General ranking structure

**Figure 1:**
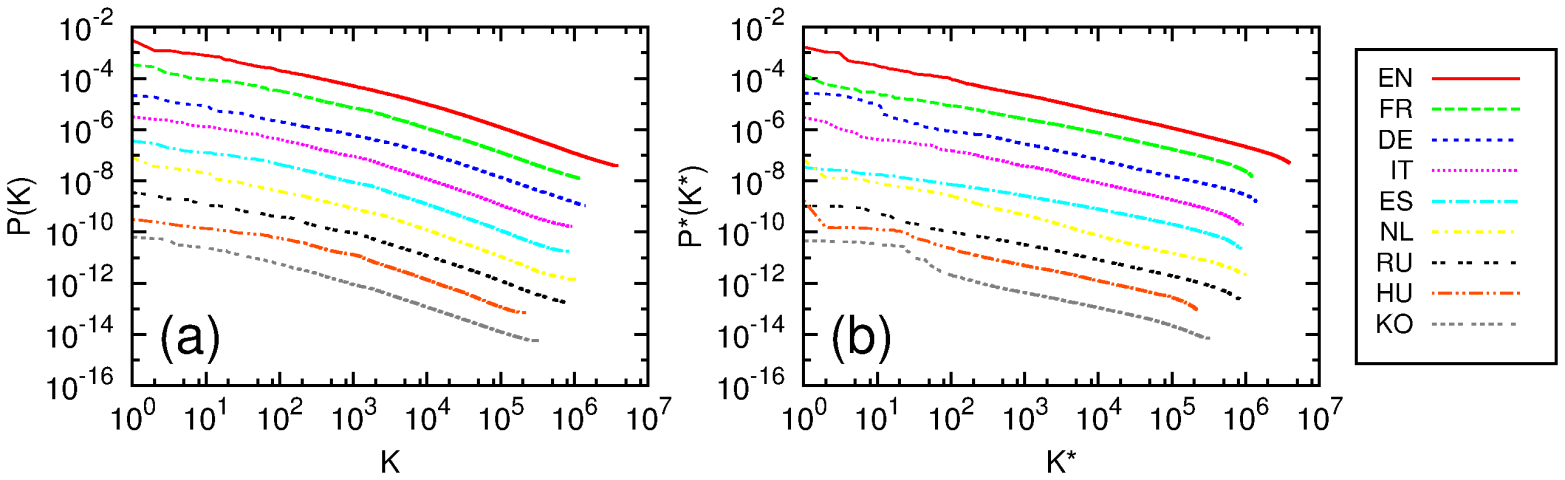
PageRank probability 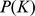
 **as function of PageRank index** 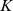 **(a) and CheiRank probability** 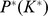 **as function of CheiRank index** 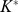 **(b).** For a better visualization each PageRank 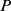 and CheiRank 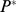 curve is shifted down by a factor 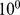 (EN), 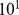 (FR), 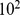 (DE), 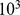 (IT), 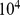 (ES), 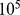 (NL), 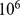 (RU), 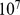 (HU), 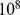 (KO).

We calculate PageRank and CheiRank probabilities and indexes for all networks of considered Wikipedia editions. The PageRank and CheiRank probabilities as functions of ranking indexes are shown in Fig. 1. The decay is compatible with an approximate algebraic decrease of a type 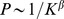, 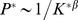 with 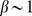 for PageRank and 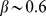 for CheiRank. These values are similar to those found for the English Wikipedia of 2009 [17]. The difference of 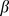 values originates from asymmetric nature between in-degree and out-degree distributions, since PageRank is based on incoming edges while CheiRank is based on outgoing edges. In-degree distribution of Wikipedia editions is broader than out-degree distribution of the same edition. Indeed, the CheiRank probability is proportional to frequency of outgoing links which has a more rapid decay compared to incoming one (see discussion in [17]). The PageRank (CheiRank) probability distributions are similar for all editions. However, the fluctuations of 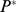 are stronger that is related to stronger fluctuations of outgoing edges [19].

The top article of PageRank is usually *USA* or the name of country of a given language (FR, RU, KO). For NL we have at the top *beetle, species, France*. The top articles of CheiRank are various listings.

**Figure 2:**
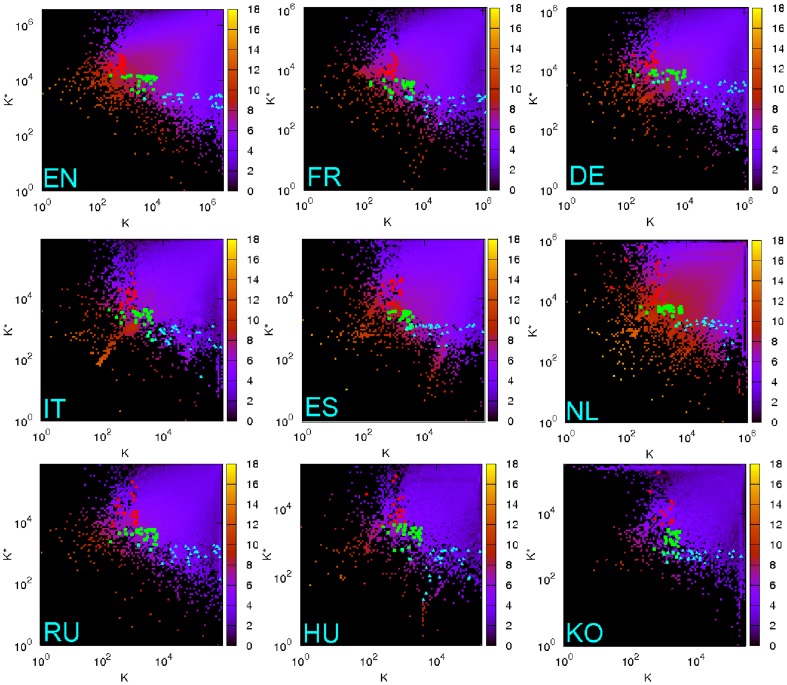
Density of Wikipedia articles in the PageRank ranking 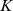
 **versus CheiRank ranking** 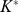 **plane for each Wikipedia edition.** The red points are top PageRank articles of persons, the green points are top 2DRank articles of persons and the cyan points are top CheiRank articles of persons. Panels show: English (top-left), French (top-center), German (top-right), Italian (middle-left), Spanish (middle-center), Dutch (middle-left), Russian (bottom-left), Hungarian (bottom-center), Korean (bottom-right). Color bars shown natural logarithm of density, changing from minimal nonzero density (dark) to maximal one (white), zero density is shown by black.

Since each article has its PageRank ranking 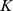 and CheiRank ranking 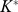, we can assign two dimensional coordinates to all the articles. Fig. 2 shows the density of articles in the two dimensional plane 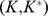 for each Wikipedia edition. The density is computed for 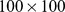 logarithmically equidistant cells which cover the whole plane 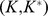. The density plot represents the locations of articles in the plane. We can observe high density of articles around line 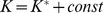 that indicates the positive correlation between PageRank and CheiRank. However, there are only a few articles within the region of top both PageRank and CheiRank indexes. We also observe the tendency that while high PageRank articles (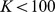) have intermediate CheiRank (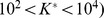, high CheiRank articles (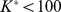) have broad PageRank rank values.

### Ranking of articles for persons

We choose top 30 articles about persons for each edition and each ranking. In Fig. 2, they are shown by red circles (PageRank), green squares (2DRank) and cyan triangles (CheiRank). We assign local ranking 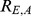 (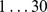) to each person in the list of top 30 persons for each edition 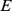 and ranking algorithm 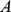. An example of 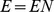 and 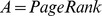 are given in Table 2.

**Table 2:** Example of list of top 10 persons by PageRank for English Wikipedia with their field of activity and native language.

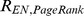	Person	Field	Culture	Locality
1	Napoleon	Politics	FR	Non-local
2	Carl Linnaeus	Science	WR	Non-local
3	George W. Bush	Politics	EN	Local
4	Barack Obama	Politics	EN	Local
5	Elizabeth II	Politics	EN	Local
6	Jesus	Religion	WR	Non-local
7	William Shakespeare	Art	EN	Local
8	Aristotle	Science	WR	Non-local
9	Adolf Hitler	Politics	DE	Non-local
10	Bill Clinton	Politics	EN	Local

**Figure 3:**
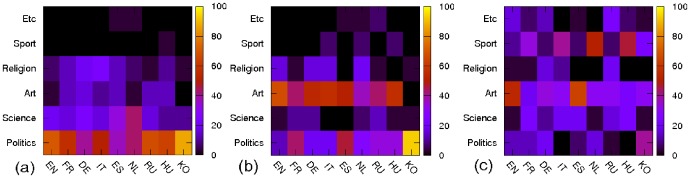
Distribution of top 30 persons in each rank over activity fields for each Wikipedia edition. Panels correspond to (a) PageRank, (b) 2DRank, (3) CheiRank. The color bar shows the values in percents.

From the lists of top persons, we identify the “fields” of activity for each top 30 rank person in which he/she is active on. We categorize six activity fields - politics, art, science, religion, sport and etc (here “etc” includes all other activities). As shown in Fig. 3, for PageRank, politics is dominant and science is secondarily dominant. The only exception is Dutch where science is the almost dominant activity field (politics has the same number of points). In case of 2DRank, art becomes dominant and politics is secondarily dominant. In case of CheiRank, art and sport are dominant fields. Thus for example, in CheiRank top 30 list we find astronomers who discovered a lot of asteroids, e.g. Karl Wilhelm Reinmuth (4th position in RU and 7th in DE), who was a prolific discoverer of about 400 of them. As a result, his article contains a long listing of asteroids discovered by him giving him a high CheiRank.

The change of activity priority for different ranks is due to the different balance between incoming and outgoing links there. Usually the politicians are well known for a broad public, hence, the articles about politicians are pointed by many articles. However, the articles about politician are not very communicative since they rarely point to other articles. In contrast, articles about persons in other fields like science, art and sport are more communicative because of listings of insects, planets, asteroids they discovered, or listings of song albums or sport competitions they gain.

Next we investigate distributions over “cultures” to which persons belong. We determined the culture of person based on the language the person mainly used (mainly native language). We consider 10 culture categories - EN, FR, DE, IT, ES, NL, RU, HU, KO and WR. Here “WR” category represents all other cultures which do not belong to considered 9 Wikipedia editions. Comparing with the culture of persons at various editions, we can assign “locality” to each 30 top rank persons for a given Wikipedia edition and ranking algorithm. For example, as shown in Table 2, *George W. Bush* belongs to “Politics”, “English” and “Local” for English Wikipedia and PageRank, while *Jesus* belongs to “Religion”, “World” WR and “Non-local”.

**Figure 4:**
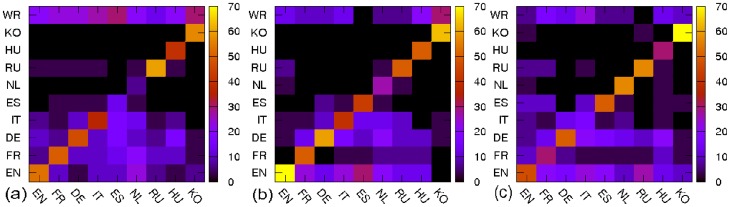
Distributions of top 30 persons over different cultures corresponding to Wikipedia editions, “WR” category represents all other cultures which do not belong to considered 9 Wikipedia editions. Panels show ranking by (a) PageRank, (b) 2DRank, (3) CheiRank. The color bar shows the values in percents.

As shown in Fig. 4, regardless of ranking algorithms, main part of top 30 ranking persons of each edition belong to the culture of the edition (usually about 50%). For example, high PageRank persons in English Wikipedia are mainly English (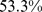). This corresponds to the self-focusing effect discussed in [6]. It is notable that top ranking persons in Korean Wikipedia are not only mainly Korean (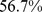) but also the most top ranking non Korean persons in Korean Wikipedia are Chinese and Japanese (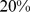). Although there is a strong tendency that each edition favors its own persons, there is also overlap between editions. For PageRank, on average, 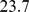 percent of top persons are overlapping while for CheiRank , the overlap is quite low, only 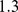 percent. For 2DRank, the overlap is 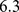 percent. The overlap of list of top persons implies the existence of cross-cultural ‘heroes’.

To understand the difference between local and non-local top persons for each edition quantitatively, we consider the PageRank case because it has a large fraction of non-local top persons. From Eq. (2), a citing article 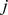 contributes 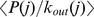 to PageRank of a node 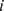. So the PageRank 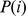 can be high if the node 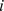 has many incoming links from citing articles 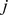 or it has incoming links from high PageRank nodes 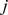 with low out-degree 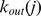. Thus we can identify origin of each top person's PageRank using the average PageRank contribution 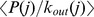 by nodes 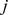 to person 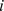 and average number of incoming edges (in-degree) 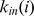 of person 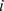 .

As represented in Table 3, considering median, local top persons have more incoming links than non-local top persons but the PageRank contribution of the corresponding links are lower than links of non-local top persons. This indicates that local top persons are cited more than non-local top persons but non-local top persons are cited more high weighted links (i.e. cited by important articles or by articles which don't have many citing links).

**Table 3:** PageRank contribution per link and in-degree of PageRank local and non-local heroes 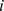 for each edition.

Edition	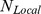	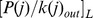		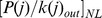	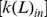		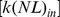
EN	16	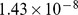	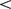	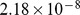	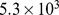	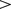	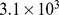
FR	15	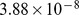	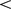	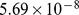	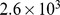	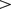	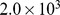
DE	14	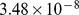	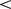	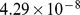	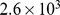	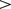	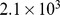
IT	11	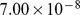	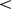	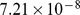	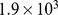	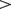	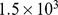
ES	4	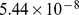	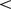	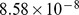	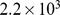	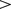	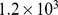
NL	2	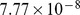	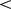	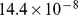	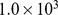	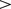	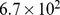
RU	18	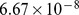	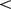	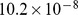	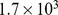	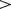	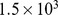
HU	12	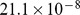	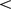	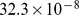	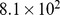	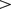	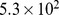
KO	17	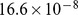	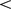	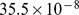	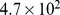	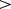	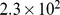

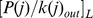
 and 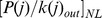 are median PageRank contribution of a local hero 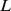 and non-local hero 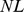 by a article 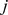 which cites local heroes 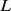 and non-local heroes 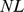 respectively. 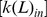 and 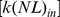 are median number of in-degree 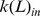 and 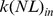 of local hero 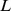 and non-local hero 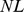, respectively. 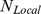 is number local heroes in given edition.

### Global and local heroes

Based on cultural dependency on rankings of persons, we can identify global and local heroes in the considered Wikipedia editions. However, for CheiRank the overlap is very low and our statistics is not sufficient for selection of global heroes. Hence we consider only PageRank and 2DRank cases. We determine the local heroes for each ranking and for each edition as top persons of the given ranking who belongs to the same culture as the edition. Top 3 local heroes for each ranking and each edition are represented in Table 4 (PageRank), Table 5 (CheiRank) and Table 6 (2DRank), respectively.

**Table 4:** List of local heroes by PageRank for each Wikipedia edition.

Edition	1st	2nd	3rd
EN	George W. Bush	Barack Obama	Elizabeth II
FR	Napoleon	Louis XIV of France	Charles de Gaulle
DE	Adolf Hitler	Martin Luther	Immanuel Kant
IT	Augustus	Dante Alighieri	Julius Caesar
ES	Charles V, Holy Roman Emperor	Philip II of Spain	Francisco Franco
NL	William I of the Netherlands	Beatrix of the Netherlands	William the Silent
RU	Peter the Great	Joseph Stalin	Alexander Pushkin
HU	Matthias Corvinus	Szentágothai János	Stephen I of Hungary
KO	Gojong of the Korean Empire	Sejong the Great	Park Chung-hee

All names are represented by article titles in English Wikipedia. Here “William the Silent” is the third local hero in Dutch Wikipedia but he is out of top 30 persons.

**Table 5:** List of local heroes by CheiRank for each Wikipedia edition.

Edition	1st	2nd	3rd
EN	C. H. Vijayashankar	Matt Kelley	William Shakespeare (inventor)
FR	Jacques Davy Duperron	Jean Baptiste Eblé	Marie-Magdeleine Aymé de La Chevrelière
DE	Harry Pepl	Marc Zwiebler	Eugen Richter
IT	Nduccio	Vincenzo Olivieri	Mina (singer)
ES	Che Guevara	Arturo Mercado	Francisco Goya
NL	Hans Renders	Julian Jenner	Marten Toonder
RU	Aleksander Vladimirovich Sotnik	Aleksei Aleksandrovich Bobrinsky	Boris Grebenshchikov
HU	Csernus Imre	Kati Kovács	Pléh Csaba
KO	Lee Jong-wook (baseball)	Kim Dae-jung	Kim Kyu-sik

All names are represented by article titles in English Wikipedia.

**Table 6:** List of local heroes by 2DRank for each Wikipedia edition.

Edition	1st	2nd	3rd
EN	Frank Sinatra	Paul McCartney	Michael Jackson
FR	François Mitterrand	Jacques Chirac	Honoré de Balzac
DE	Adolf Hitler	Otto von Bismarck	Ludwig van Beethoven
IT	Giusppe Garibaldi	Raphael	Benito Mussolini
ES	Simón Bolívar	Francisco Goya	Fidel Castro
NL	Albert II of Belgium	Johan Cruyff	Rembrandt
RU	Dmitri Mendeleev	Peter the Great	Yaroslav the Wise
HU	Stephen I of Hungary	Sándor Petöfi	Franz Liszt
KO	Gojong of the Korean Empire	Sejong the Great	Park Chung-hee

All names are represented by article titles in English Wikipedia.

In order to identify the global heroes, we define ranking score 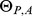 for each person 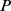 and each ranking algorithm 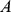. Since every person in the top person list has relative ranking 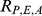 for each Wikipedia edition 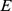 and ranking algorithm 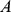 (For instance, in Table 2, 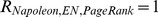). The ranking score 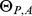 of a person 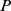 is give by
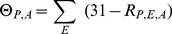
(5)


According to this definition, a person who appears more often in the lists of editions and has top ranking in the list gets high ranking score. We sort this ranking score for each algorithm. In this way obtain a list of global heroes for each algorithm. The result is shown in Table 7. Napoleon is the 1st global hero by PageRank and Micheal Jackson is the 1st global hero by 2DRank.

**Table 7:** List of global heroes by PageRank and 2DRank for all 9 Wikipedia editions.

Rank	PageRank global heroes	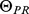	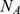	2DRank global heroes	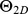	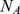
1st	Napoleon	259	9	Micheal Jackson	119	5
2nd	Jesus	239	9	Adolf Hitler	93	6
3rd	Carl Linnaeus	235	8	Julius Caesar	85	5
4th	Aristotle	228	9	Pope Benedict XVI	80	4
5th	Adolf Hitler	200	9	Wolfgang Amadeus Mozart	75	5
6th	Julius Caesar	161	8	Pope John Paul II	71	4
7th	Plato	119	6	Ludwig van Beethoven	69	4
8th	Charlemagne	111	8	Bob Dylan	66	4
9th	William Shakespeare	110	7	William Shakespeare	57	3
10th	Pope John Paul II	108	6	Alexander the Great	56	3

All names are represented by article titles in English Wikipedia. Here, 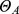 is the ranking score of the algorithm 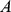 (5); 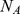 is the number of appearances of a given person in the top 30 rank for all editions.

### Network of cultures

**Figure 5:**
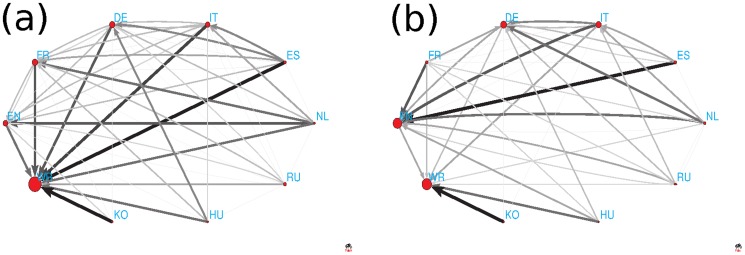
Network of cultures obtained from 9 Wikipedia languages and the remaining world (WR) selecting 30 top persons of PageRank (a) and 2DRank (b) in each culture. The link width and darkness are proportional to a number of foreign persons quoted in top 30 of a given culture, the link direction goes from a given culture to cultures of quoted foreign persons, quotations inside cultures are not considered. The size of nodes is proportional to their PageRank.

**Figure 6:**
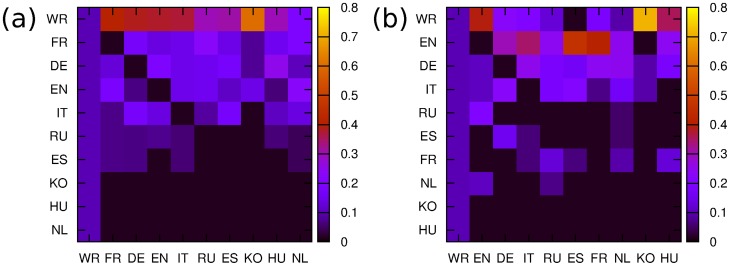
Google matrix of network of cultures from **Fig. 5**, shown respectively for panels 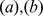
 **.** The matrix elements 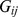 are shown by color at the damping factor 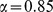, index 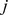 is chosen as the PageRank index 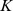 of PageRank vector so that the top cultures with 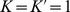 are located at the top left corner of the matrix.

To characterize the entanglement and interlinking of cultures we use the data of Fig. 4 and from them construct the network of cultures. The image of networks obtained from top 30 persons of PageRank and 2DRank listings are shown in Fig. 5 (we do not consider CheiRank case due to small overlap of persons resulting in a small data statistics). The weight of directed Markov transition, or number of links, from a culture 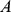 to a culture 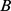 is given by a number of persons of a given culture 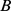 (e.g FR) appearing in the list of top 30 persons of PageRank (or 2DRank) in a given culture 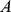 (e.g. EN). Thus e.g. for transition from EN to FR in PageRank we find 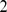 links (2 French persons in PageRank top 30 persons of English Wikipedia); for transition from FR to EN in PageRank we have 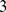 links (3 English persons in PageRank top 30 persons of French Wikipedia). The transitions inside each culture (persons of the same language as language edition) are omitted since we are analyzing the interlinks between cultures. Then the Google matrix of cultures is constructed by the standard rule for the directed networks: all links are treated democratically with the same weight, sum of links in each column is renormalized to unity, 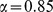. Even if this network has only 10 nodes we still can find for it PageRank and CheiRank probabilities 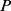 and 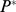 and corresponding indexes 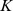 and 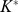. The matrix elements of 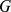 matrix, written in order of index 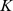, are shown in Fig. 6 for the corresponding networks of cultures presented in Fig. 5. We note that we consider all cultures on equal democratic grounds.

**Figure 7:**
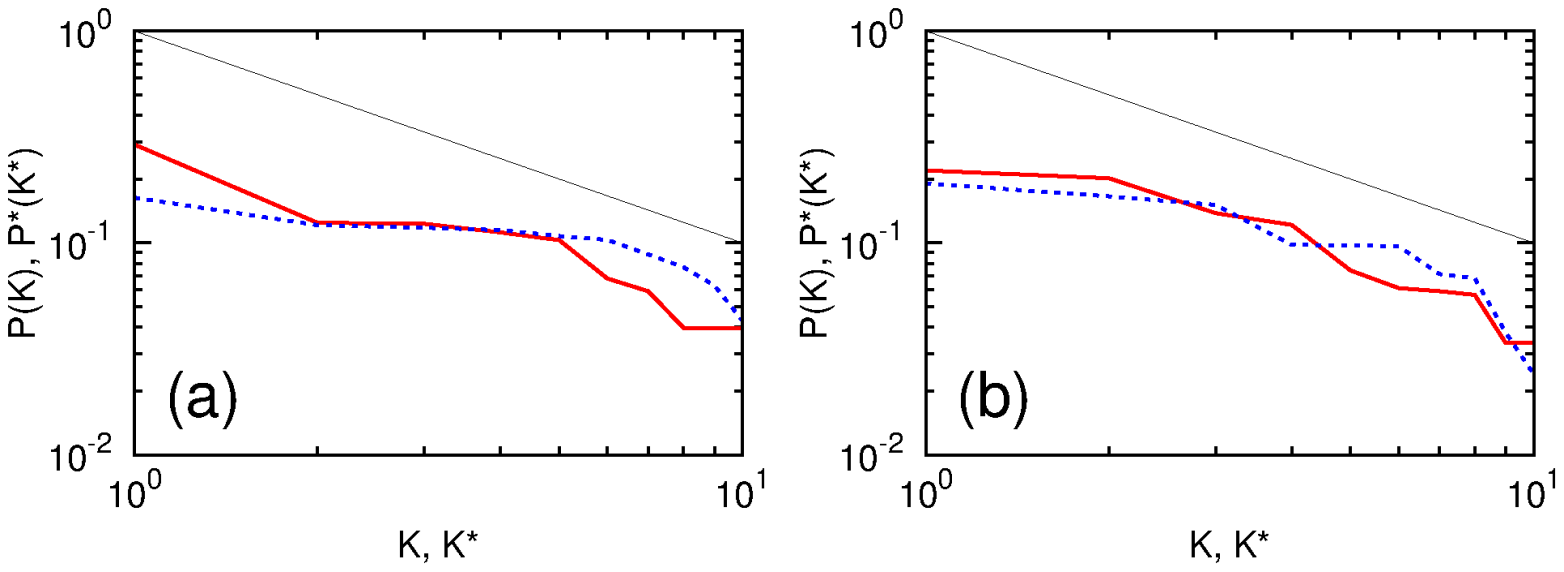
Dependence of probabilities of PageRank 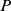
 **(red) and CheiRank** 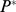 **(blue) on corresponding indexes** 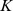 **and** 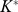 **.** The probabilities are obtained from the network and Google matrix of cultures shown in Fig. 5 and Fig. 6 for corresponding panels 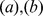. The straight lines indicate the Zipf law 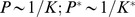.

The decays of PageRank and CheiRank probabilities with the indexes 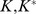 are shown in Fig. 7 for the culture networks of Fig. 5. On a first glance a power decay like the Zipf law [27] 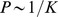 looks to be satisfactory. The formal power law fit 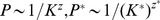, done in log–log-scale for 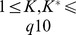, gives the exponents 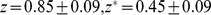 (Fig. 7a), 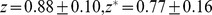 (Fig. 7b). However, the error bars for these fits are relatively large. Also other statistical tests (e.g. the Kolmogorov-Smirnov test, see details in [28]) give low statistical accuracy (e.g. statistical probability 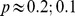 and 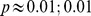 for exponents 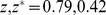 and 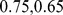 in Fig. 7a and Fig. 7b respectively). It is clear that 10 cultures is too small to have a good statistical accuracy. Thus, a larger number of cultures should be used to check the validity of the generalized Zipf law with a certain exponent. We make a conjecture that the Zipf law with the generalized exponents 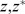 will work in a better way for a larger number of multilingual Wikipedia editions which now have about 250 languages.

**Figure 8:**
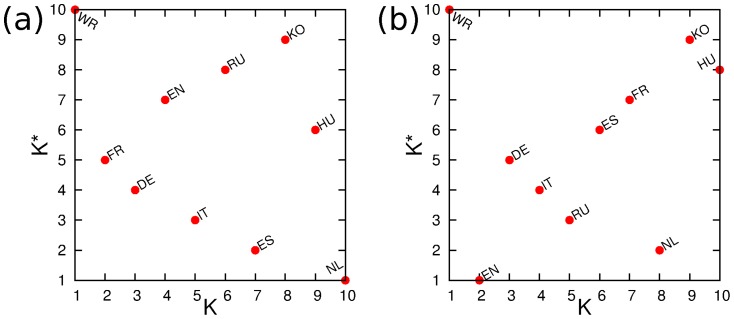
PageRank versus CheiRank plane of cultures with corresponding indexes 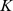
 **and** 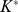 **obtained from the network of cultures for corresponding panels** 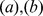 **.**

The distributions of cultures on the PageRank - CheiRank plane 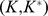 are shown in Fig. 8. For the network of cultures constructed from top 30 PageRank persons we obtain the following ranking. The node WR is located at the top PageRank 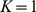 and it stays at the last CheiRank position 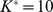. This happens due to the fact that such persons as *Carl Linnaeus, Jesus, Aristotle, Plato, Alexander the Great, Muhammad* are not native for our 9 Wikipedia editions so that we have many nodes pointing to WR node, while WR has no outgoing links. The next node in PageRank is FR node at 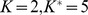, then DE node at 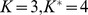 and only then we find EN node at 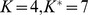. The node EN is not at all at top PageRank positions since it has many American politicians that does not count for links between cultures. After the world WR the top position is taken by French (FR) and then German (DE) cultures which have strong links inside the continental Europe.

However, the ranking is drastically changed when we consider top 30 2DRank persons. Here, the dominant role is played by art and science with singers, artists and scientists. The world WR here remains at the same position at 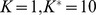 but then we obtain English EN (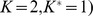 and German DE (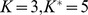) cultures while FR is moved to 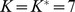.

## Discussion

We investigated cross-cultural diversity of Wikipedia via ranking of Wikipedia articles. Even if the used ranking algorithms are purely based on network structure of Wikipedia articles, we find cultural distinctions and entanglement of cultures obtained from the multilingual editions of Wikipedia.

In particular, we analyze raking of articles about persons and identify activity field of persons and cultures to which persons belong. Politics is dominant in top PageRank persons, art is dominant in top 2DRank persons and in top CheiRank persons art and sport are dominant. We find that each Wikipedia edition favors its own persons, who have same cultural background, but there are also cross-cultural non-local heroes, and even “global heroes”. We establish that local heroes are cited more often but non-local heroes on average are cited by more important articles.

Attributing top persons of the ranking list to different cultures we construct the network of cultures and characterize entanglement of cultures on the basis of Google matrix analysis of this directed network.

We considered only 9 Wikipedia editions selecting top 30 persons in a “manual” style. It would be useful to analyze a larger number of editions using an automatic computerized selection of persons from prefabricated listing in many languages developing lines discussed in [14]. This will allow to analyze a large number of persons improving the statistical accuracy of links between different cultures.

The importance of understanding of cultural diversity in globalized world is growing. Our computational, data driven approach can provide a quantitative and efficient way to understand diversity of cultures by using data created by millions of Wikipedia users. We believe that our results shed a new light on how organized interactions and links between different cultures.

## Supporting Information

File S1 Presents Figures S1, S2, S3 in SI file showing comparison between probability distributions over activity fields and language for top 30 and 100 persons for EN, IT, NK respectively; tables S1, S2, … S27 in SI file showing top 30 persons in PageRank, CheiRank and 2DRank for all 9 Wikipedia editions. All names are given in English. Supplementary methods, tables, ranking lists and figures are available at http://www.quantware.ups-tlse.fr/QWLIB/wikiculturenetwork/; data sets of 9 hyperlink networks are available at [29] by a direct request addressed to S.Vigna.
(PDF)

## References

[1] Borges JL (1962) *The Library of Babel* in *Ficciones*, Grove Press, New York

[2] Kaltenbrunner A, Laniado D (2012) *There is no deadline - time evolution of Wikipedia discussions*, Proc. of the 8th Intl. Symposium on Wikis and Open Collaboration, Wik- iSym12, Linz

[3] Torok J, Iniguez G, Yasseri T, San Miguel M, Kaski K, et al (2013) *Opinion, conflicts and consensus: modeling social dynamics in a collaborative enviroment* . Phys Rev Lett 110: 088701. 23473207 10.1103/PhysRevLett.110.088701

[4] Yasseri T, Kornai A, Kertész J (2012) *A practical approach to language complexity: a Wikipedia case study* . PLoS ONE 7: e48386. 23189130 10.1371/journal.pone.0048386 PMC3492358

[5] Brandes U, Kenis P, Lerner U, van Raaij D (2009) *Network analysis of collaboration structure in Wikipedia* Proc. 18th Intl. Conf. WWW, :731

[6] Hecht B, Gergle D (2009) *Measuring self-focus bias in community-maintained knowledge repositories* Proc. of the Fourth Intl Conf. Communities and technologies, ACM, New York :11

[7] Nemoto K, Gloor PA (2011) *Analyzing cultural differences in collaborative innovation networks by analyzing editing behavior in different-language Wikipedias* . Procedia - Social and Behavioral Sciences 26: 180.

[8] Norenzayan A (2011) *Explaining human behavioral diversity* . Science 332: 1041. 21617064 10.1126/science.1207050

[9] Gelfand MJ, Raver JL, Nishii L, Leslie LM, Lun J, et al (2011) *Differences between tight and loose cultures: a 33-nation study* . Science 332: 1100. 21617077 10.1126/science.1197754

[10] Yasseri T, Spoerri A, Graham M, Kertész J (2013) *The most controversial topics in Wikipedia: a multilingual and geographical analysis* arXiv:1305.5566 [physics.soc-ph]

[11] UNESCO World Report (2009) *Investing in cultural diversity and intercultural dialogue*, Available: http://www.unesco.org/new/en/culture/resources/report/the-unesco-world-report- on-cultural-diversity

[12] Callahan ES, Herriing SC (2011) *Cultural bias in Wikipedia content on famous persons* . Journal of the American society for information science and technology 62: 1899.

[13] Warncke-Wang M, Uduwage A, Dong Z, Riedl J (2012) *In search of the ur-Wikipedia: universality, similarity, and translation in the Wikipedia inter-language link network*, Proceedings of the Eighth Annual International Symposium on Wikis and Open Collaboration (WikiSym 2012), ACM, New York No 20

[14] Aragón P, Laniado D, Kaltenbrunner A, Volkovich Y (2012) *Biographical social networks on Wikipedia: a cross-cultural study of links that made history*, Proceedings of the Eighth Annual International Symposium on Wikis and Open Collaboration (WikiSym 2012), ACM, New York No 19; arXiv:1204.3799v2[cs.SI]

[15] Brin S, Page L (1998) The anatomy of a large-scale hypertextual Web search engine. Computer Networks and ISDN Systems 30: 107.

[16] Chepelianskii AD (2010) *Towards physical laws for software architecture* arXiv:1003.5455 [cs.SE]

[17] Zhirov AO, Zhirov OV, Shepelyansky DL (2010) *Two-dimensional ranking of Wikipedia articles* . Eur Phys J B 77: 523.

[18] Ermann L, Chepelianskii AD, Shepelyansky DL (2012) *Toward two-dimensional search engines* . J Phys A: Math Theor 45: 275101.

[19] Eom YH, Frahm KM, Bencźur A, Shepelyansky DL (2013) *Time evolution of Wikipedia network ranking* arXiv:1304.6601 [physics.soc-ph]

[20] Langville AM, Meyer CD (2006) *Google's PageRank and Beyond: The Science of Search Engine Rankings*, Princeton University Press, Princeton

[21] Brin M, Stuck G (2002) *Introduction to dynamical systems*, Cambridge Univ. Press, Cambridge, UK

[22] Ermann L, Frahm KM, Shepelyansky DL (2013) *Spectral properties of Google matrix of Wikipedia and other networks* . Eur Phys J D 86: 193.

[23] Chen P, Xie H, Maslov S, Redner S (2007) *Finding scientific gems with Googleś PageRank algorithm* . Jour Informetrics 1: 8.

[24] Kwak H, Lee C, Park H, Moon S (2010) *What is Twitter, a social network or a news media?*, Proc. 19th Int. Conf. WWW2010, ACM, New York :591

[25] Kandiah V, Shepelyansky DL (2013) *Google matrix analysis of DNA sequences* . PLoS ONE 8 ((5)) e61519. 23671568 10.1371/journal.pone.0061519 PMC3650020

[26] Ermann L, Shepelyansky DL (2011) *Google matrix of the world trade network*, Acta Physica Polonica A 120 ((6A)): , A158

[27] Zipf GK (1949) *Human behavior and the principle of least effort*, Addison-Wesley, Boston

[28] Clauset A, Shalizi CR, Newman MEJ (2009) *Power-law distributions in empirical data* . SIAM Rev 51 ((4)) 661.

[29] Personal website of Sebastiano Vigna. Available: http://vigna.dsi.unimi.it/. Accessed 2013 Jun 26.

