# Supplementary material for: Highlighting Entanglement of Cultures via Ranking of Multilingual Wikipedia Articles
Source: PLoS One. 2013 Oct 3;8(10):e74554. doi: 10.1371/journal.pone.0074554 (PMC3789750; doi:10.1371/journal.pone.0074554)
Supplement: File S1 — Presents Figures S1, S2, S3 in SI file showing comparison between probability distributions over activity fields and language for top 30 and 100 persons for EN, IT, NK respectively; tables S1, S2, … S27 in SI file showing top 30 persons in PageRank, CheiRank and 2DRank for all 9 Wikipedia editions. All names are given in English. Supplementary methods, tables, ranking lists and figures are available at http://www.quantware.ups-tlse.fr/QWLIB/wikiculturenetwork/; data sets of 9 hyperlink networks are available at [29] by a direct request addressed to S.Vigna. (PDF) [file pone.0074554.s001.pdf]

SUPPORTING INFORMATION FOR: Highlighting entanglement of cultures via ranking of multilingual Wikipedia articles

Young-Ho Eom[1], Dima L. Shepelyansky[1,*]

1 *Laboratoire de Physique Théorique du CNRS, IRSAMC, Université de Toulouse, UPS, F-31062 Toulouse, France*

* Webpage: www.quantware.ups-tlse.fr/dima

1 Additional data

Supplementary methods, tables, ranking lists and figures are available at
http://www.quantware.ups-tlse.fr/QWLIB/wikiculturenetwork/;
data sets of 9 hyperlink networks are available at
http://vigna.dsi.unimi.it/
by a direct request addressed to S.Vigna.

Here we present additional figures and tables for the main part of the paper.

Figures S1, S2, S3 show comparison between probability distributions over activity fields and language for top 30 and 100 persons for EN, IT, NK respectively.

Tables show top 30 persons in PageRank, CheiRank and 2DRank for all 9 Wikipedia editions. All names are given in English.

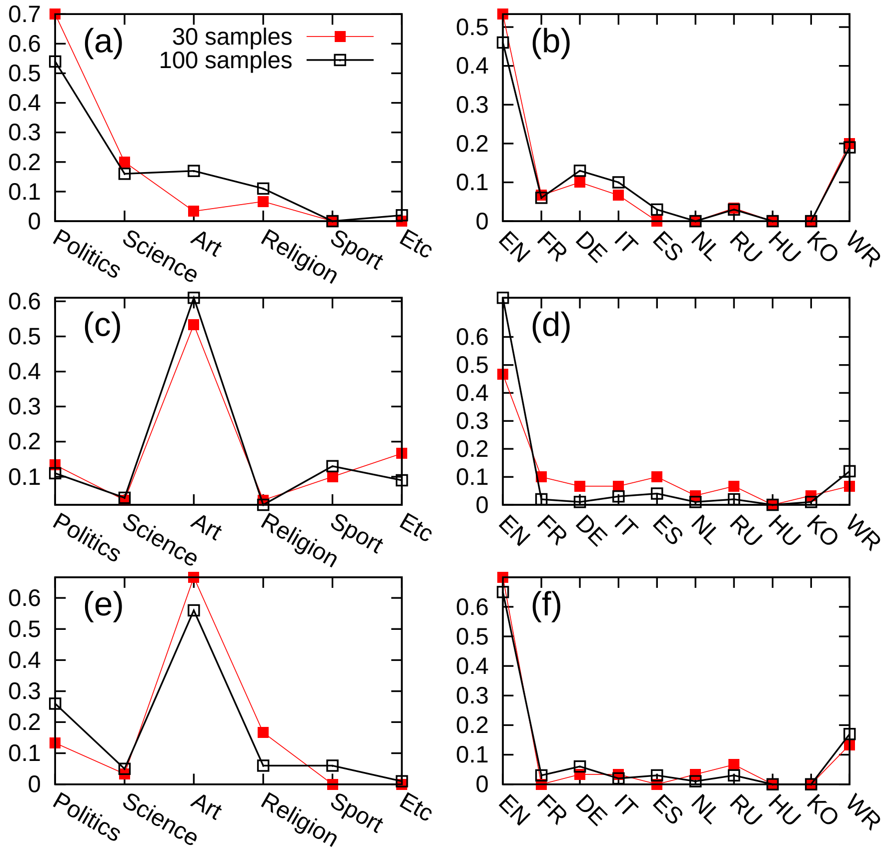

Figure S1: Probability distributions of activity fields and languages of top 30 persons and top 100 persons in English Wikipedia EN (total probability is normalized to unity): (a) Distribution of activity fields of PageRank top persons (b) Distribution of langauge of PageRank top persons. (c) Distribution of activity fields of CheiRank top persons (d) Distribution of langauge of CheiRank top persons. (e) Distribution of activity fields of 2DRank top persons (f) Distribution of langauge of 2DRank top persons.

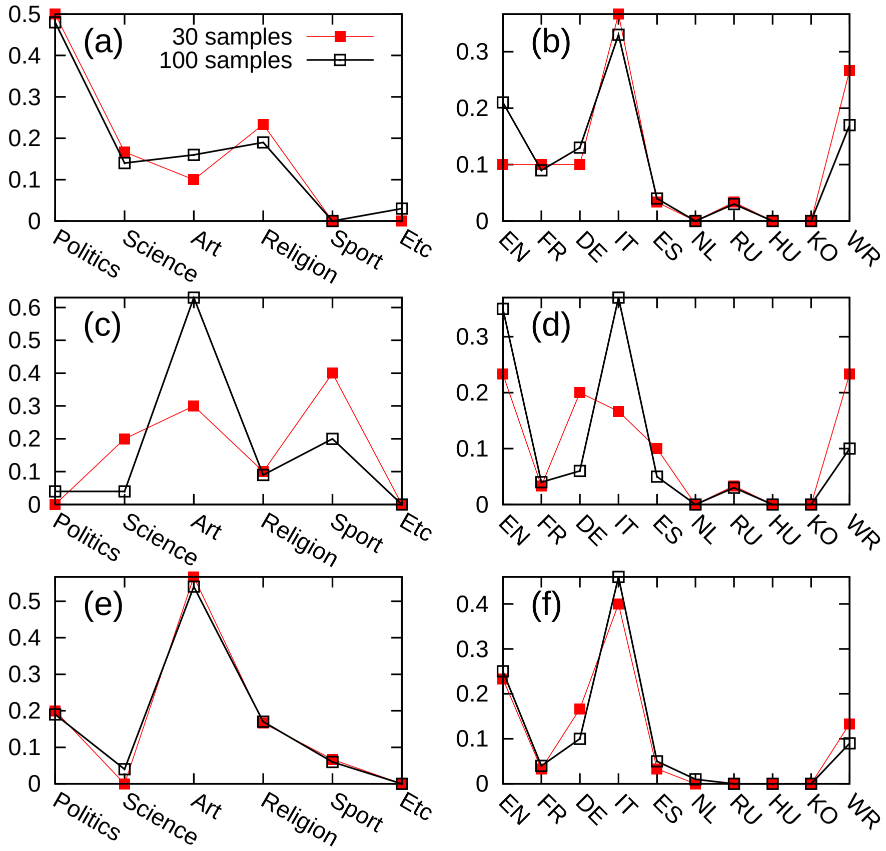

Figure S2: Same as in Fig.SI1 for Italian Wikipedia IT.

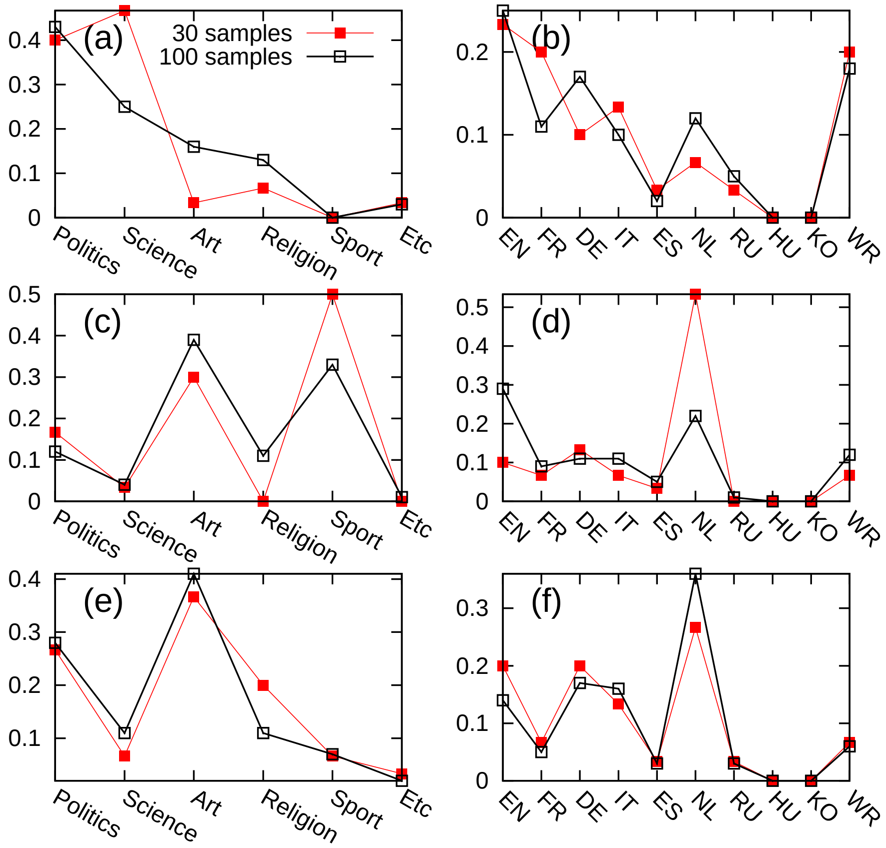

Figure S3: Same as in Fig.SI1 for Dutch Wikipedia NL.

Table S1: Top 30 persons by PageRank for English Wikipedia with their field of activity and native language.

$R_{EN,PageRank}$	Person	Field	Culture
1	Napoleon	Politics	FR
2	Carl Linnaeus	Science	WR
3	George W. Bush	Politics	EN
4	Barack Obama	Politics	EN
5	Elizabeth II	Politics	EN
6	Jesus	Religion	WR
7	William Shakespeare	Art	EN
8	Aristotle	Science	WR
9	Adolf Hitler	Politics	DE
10	Bill Clinton	Politics	EN
11	Franklin D. Roosevelt	Politics	EN
12	Ronald Reagan	Politics	EN
13	George Washington	Politics	EN
14	Plato	Science	WR
15	Richard Nixon	Politics	EN
16	Abraham Lincoln	Politics	EN
17	Joseph Stalin	Politics	RU
18	Winston Churchill	Politics	EN
19	John F. Kennedy	Politics	EN
20	Henry VIII of England	Politics	EN
21	Muhammad	Religion	WR
22	Thomas Jefferson	Politics	EN
23	Albert Einstein	Science	DE
24	Alexander the Great	Politics	WR
25	Augustus	Politics	IT
26	Charlemagne	Politics	FR
27	Karl Marx	Science	DE
28	Charles Darwin	Science	EN
29	Elizabeth I of England	Politics	EN
30	Julius Caesar	Politics	IT

Table S2: Top 30 persons by 2DRank for English Wikipedia with their field of activity and native language.

$R_{EN,2DRank}$	Person	Field	Culture
1	Frank Sinatra	Art	EN
2	Paul McCartney	Art	EN
3	Michael Jackson	Art	EN
4	Steven Spielberg	Art	EN
5	Pope Pius XII	Religion	IT
6	Vladimir Putin	Politics	RU
7	Mariah Carey	Art	EN
8	John Kerry	Politics	EN
9	Isaac Asimov	Art	EN
10	Stephen King	Art	EN
11	Dolly Parton	Art	EN
12	Prince (musician)	Art	EN
13	Robert Brown (botanist)	Science	EN
14	Vincent van Gogh	Art	NL
15	Lady Gaga	Art	EN
16	Beyoncé Knowles	Art	EN
17	Pope John Paul II	Religion	WR
18	Lord Byron	Art	EN
19	Muhammad	Religion	WR
20	Johnny Cash	Art	EN
21	Alice Cooper	Art	EN
22	Catherine the Great	Politics	RU
23	14th Dalai Lama	Religion	WR
24	Christina Aguilera	Art	EN
25	Marilyn Monroe	Art	EN
26	David Bowie	Art	EN
27	John McCain	Politics	EN
28	Bob Dylan	Art	EN
29	Johann Sebastian Bach	Art	DE
30	Jesus	Religion	WR

Table S2: Top 30 persons by CheiRank for English Wikipedia with their field of activity and native language.

$R_{EN,CheiRank}$	Person	Field	Culture
1	Roger Calmel	Art	FR
2	C. H. Vijayashankar	Politics	EN
3	Matt Kelley	ETC	EN
4	Alberto Cavallari	ETC	IT
5	Yury Chernavsky	Art	RU
6	William Shakespeare (inventor)	ETC	EN
7	Kelly Clarkson	Art	EN
8	Park Ji-Sung	Sport	KO
9	Mithun Chakraborty	Art	EN
10	Olga Sedakova	Sport	RU
11	Sara García	Art	ES
12	Pope Pius XII	Religion	IT
13	Andy Kerr	Politics	EN
14	Joe-Max Moore	Sport	EN
15	Josef Kemr	Art	WR
16	Darius Milhaud	Art	FR
17	Jan Crull, Jr.	ETC	EN
18	Farshad Fotouhi	Science	EN
19	Swaroop Kanchi	Art	EN
20	Jacques Lancelot	Art	FR
21	František Martin Pecháček	Art	DE
22	George Stephanekoulosech	ETC	EN
23	Chano Urueta	Art	ES
24	Franz Pecháček	Art	DE
25	Nicolae Iorga	Politics	WR
26	Arnold Houbraken	Art	NL
27	August Derleth	Art	EN
28	Javier Solana	Politics	ES
29	Drew Barrymore	Art	EN
30	Kevin Bloody Wilson	Art	EN

Table S4: Top 30 persons by PageRank for French Wikipedia with their field of activity and native language.

$R_{FR,PageRank}$	Person	Field	Culture
1	Napoleon	Politics	FR
2	Carl Linnaeus	Science	WR
3	Louis XIV of France	Politics	FR
4	Jesus	Religion	WR
5	Aristotle	Science	WR
6	Julius Caesar	Politics	IT
7	Charles de Gaulle	Politics	FR
8	Pope John Paul II	Religion	WR
9	Adolf Hitler	Politics	DE
10	Plato	Science	WR
11	Charlemagne	Politics	FR
12	Joseph Stalin	Politics	RU
13	Charles V, Holy Roman Emperor	Politics	ES
14	Napoleon III	Politics	FR
15	Nicolas Sarkozy	Politics	FR
16	Franois Mitterrand	Politics	FR
17	Victor Hugo	Art	FR
18	Jacques Chirac	Politics	FR
19	Honore de Balzac	Art	FR
20	Mary (mother of Jesus)	Religion	WR
21	Voltaire	Art	FR
22	George W. Bush	Politics	EN
23	Elizabeth II	Politics	EN
24	Muhammad	Religion	WR
25	Francis I of France	Politics	FR
26	William Shakespeare	Art	EN
27	Louis XVI of France	Politics	FR
28	Rene Descartes	Science	FR
29	Karl Marx	Science	DE
30	Louis XV of France	Politics	FR

Table S5: Top 30 persons by 2DRank for French Wikipedia with their field of activity and native language.

$R_{FR,2DRank}$	Person	Field	Culture
1	Franois Mitterrand	Politics	FR
2	Jacques Chirac	Politics	FR
3	Honore de Balzac	Art	FR
4	Nicolas Sarkozy	Politics	FR
5	Napoleon III	Politics	FR
6	Otto von Bismarck	Politics	DE
7	Michael Jackson	Art	EN
8	Adolf Hitler	Politics	DE
9	Ludwig van Beethoven	Art	DE
10	Johnny Hallyday	Art	FR
11	Napoleon	Politics	FR
12	Leonardo da Vinci	Art	IT
13	Jules Verne	Art	FR
14	Jacques-Louis David	Art	FR
15	Thomas Jefferson	Politics	EN
16	Sigmund Freud	Science	DE
17	Madonna (entertainer)	Art	EN
18	Serge Gainsbourg	Art	FR
19	14th Dalai Lama	Religion	WR
20	Alfred Hitchcock	Art	EN
21	Georges Clemenceau	Politics	FR
22	Carl Linnaeus	Science	WR
23	Steven Spielberg	Art	EN
24	J. R. R. Tolkien	Art	EN
25	Arthur Rimbaud	Art	FR
26	Charles Darwin	Science	EN
27	Maximilien de Robespierre	Politics	FR
28	Nelson Mandela	Politics	WR
29	Henry IV of France	Politics	FR
30	Charles de Gaulle	Politics	FR

Table S6: Top 30 persons by CheiRank for French Wikipedia with their field of activity and native language.

$R_{FR,CheiRank}$	Person	Field	Culture
1	John Douglas Lynch	Science	EN
2	Roger Federer	Sport	DE
3	Richard Upjohn Light	Science	EN
4	Jacques Davy Duperron	Art	FR
5	Rafael Nadal	Sport	ES
6	Martina Navratilova	Sport	EN
7	Michael Ilmari Saaristo	Science	WR
8	Kevin Bacon	Art	EN
9	Jean Baptiste Eble	Etc	FR
10	Marie-Magdeleine Ayme de La Chevreliere	Politics	FR
11	Nataliya Pyhyda	Sport	RU
12	Max Wolf	Science	DE
13	14th Dalai Lama	Religion	WR
14	Francoise Hardy	Art	FR
15	Ghislaine N. H. Sathoud	Etc	FR
16	Frank Glaw	Science	DE
17	Johnny Hallyday	Art	FR
18	Juan A. Rivero	Science	ES
19	Valentino Rossi	Sport	IT
20	Sheila (singer)	Art	FR
21	Franois Mitterrand	Politics	FR
22	Christopher Walken	Art	EN
23	Georges Clemenceau	Politics	FR
24	Elgin Loren Elwais	Sport	WR
25	Otto von Bismarck	Politics	DE
26	Edward Drinker Cope	Science	EN
27	Rashidi Yekini	Sport	WR
28	Tofiri Kibuuka	Sport	WR
29	Paola Espinosa	Sport	ES
30	Aksana Drahun	Sport	RU

Table S7: Top 30 persons by PageRank for German Wikipedia with their field of activity and native language.

$R_{DE,PageRank}$	Person	Field	Culture
1	Napoleon	Politics	FR
2	Carl Linnaeus	Science	WR
3	Adolf Hitler	Politics	DE
4	Aristotle	Science	WR
5	Johann Wolfgang von Goethe	Art	DE
6	Martin Luther	Religion	DE
7	Jesus	Religion	WR
8	Immanuel Kant	Science	DE
9	Charlemagne	Politics	FR
10	Plato	Science	WR
11	Pope John Paul II	Religion	WR
12	Karl Marx	Science	DE
13	Julius Caesar	Politics	IT
14	Augustus	Politics	IT
15	Louis XIV of France	Politics	FR
16	Friedrich Schiller	Art	DE
17	Wolfgang Amadeus Mozart	Art	DE
18	William Shakespeare	Art	EN
19	Josef Stalin	Politics	RU
20	Pope Benedict XVI	Religion	DE
21	Otto von Bismarck	Politics	DE
22	Cicero	Politics	IT
23	Wilhelm II, German Emperor	Politics	DE
24	Johann Sebastian Bach	Art	DE
25	Max Weber	Science	DE
26	Charles V, Holy Roman Emperor	Politics	ES
27	Frederick the Great	Politics	DE
28	Georg Wilhelm Friedrich Hegel	Science	DE
29	Mary (mother of Jesus)	Religion	WR
30	Augustine of Hippo	Religion	WR

Table S8: Top 30 persons by 2DRank for German Wikipedia with their field of activity and native language.

$R_{DE,2DRank}$	Person	Field	Culture
1	Adolf Hitler	Politics	DE
2	Otto von Bismarck	Politics	DE
3	Pope Paul VI	Religion	IT
4	Ludwig van Beethoven	Art	DE
5	Franz Kafka	Art	DE
6	George Frideric Handel	Art	DE
7	Gerhart Hauptmann	Art	DE
8	Bob Dylan	Art	EN
9	Johann Sebastian Bach	Art	DE
10	Alexander the Great	Politics	WR
11	Martin Luther	Religion	DE
12	Julius Caesar	Politics	IT
13	Joseph Beuys	Art	DE
14	Pope Leo XIII	Religion	IT
15	Carl Friedrich Gauss	Science	DE
16	Andy Warhol	Art	EN
17	Alfred Hitchcock	Art	EN
18	Thomas Mann	Art	DE
19	John Lennon	Art	EN
20	Augustus II the Strong	Politics	DE
21	Pope Benedict XVI	Religion	DE
22	Ferdinand II of Aragon	Politics	ES
23	Arthur Schnitzler	Art	DE
24	Martin Heidegger	Science	DE
25	Albrecht Dürer	Art	DE
26	Carl Linnaeus	Science	WR
27	Pablo Picasso	Art	ES
28	Rainer Werner Fassbinder	Art	DE
29	Wolfgang Amadeus Mozart	Art	DE
30	Historical Jesus	Religion	WR

Table S9: Top 30 persons by CheiRank for German Wikipedia with their field of activity and native language.

$R_{DE,CheiRank}$	Person	Field	Culture
1	Diomede Carafa	Religion	IT
2	Harry Pepl	Art	DE
3	Marc Zwiebler	Sport	DE
4	Eugen Richter	Politics	DE
5	John of Nepomuk	Religion	WR
6	Pope Marcellus II	Religion	IT
7	Karl Wilhelm Reinmuth	Science	WR
8	Johannes Molzahn	Art	DE
9	Georges Vanier	ETC	FR
10	Arthur Willibald Königsheim	ETC	DE
11	Thomas Fitzsimons	Politics	EN
12	Nelson W. Aldrich	Politics	EN
13	Ma Jun	ETC	WR
14	Michael Psellos	Religion	WR
15	Adolf Hitler	Politics	DE
16	Edoardo Fazzioli	ETC	IT
17	Ray Knepper	Sport	EN
18	Frédéric de Lafresnaye	Science	FR
19	Joan Crawford	Art	EN
20	Stephen King	Art	EN
21	Gerhart Hauptmann	Art	DE
22	Paul Moder	Politics	DE
23	Erni Mangold	Art	DE
24	Robert Stolz	Art	DE
25	Otto von Bismarck	Politics	DE
26	Christine Holstein	Art	DE
27	Pope Paul VI	Religion	IT
28	Franz Buxbaum	Science	DE
29	Gustaf Gründgens	Art	DE
30	Ludwig van Beethoven	Art	DE

Table S10: Top 30 persons by PageRank for Italian Wikipedia with their field of activity and native language.

$R_{IT,PageRank}$	Person	Field	Culture
1	Napoleon	Politics	FR
2	Jesus	Religion	WR
3	Aristotle	Science	WR
4	Augustus	Politics	IT
5	Pope John Paul II	Religion	WR
6	Dante Alighieri	Art	IT
7	Adolf Hitler	Politics	DE
8	Julius Caesar	Politics	IT
9	Benito Mussolini	Politics	IT
10	Charlemagne	Politics	FR
11	Mary (mother of Jesus)	Religion	WR
12	Plato	Science	WR
13	Isaac Newton	Science	EN
14	Charles V, Holy Roman Emperor	Politics	ES
15	Galileo Galilei	Science	IT
16	Louis XIV of France	Politics	FR
17	Constantine the Great	Politics	IT
18	Cicero	Politics	IT
19	Alexander the Great	Politics	WR
20	Paul the Apostle	Politics	WR
21	Albert Einstein	Science	DE
22	Joseph Stalin	Politics	RU
23	George W. Bush	Politics	EN
24	Silvio Berlusconi	Politics	IT
25	William Shakespeare	Art	EN
26	Augustine of Hippo	Religion	WR
27	Pope Paul VI	Religion	IT
28	Pope Benedict XVI	Religion	DE
29	Giuseppe Garibaldi	Politics	IT
30	Leonardo da Vinci	Science	IT

Table S11: Top 30 persons by 2DRank for Italian Wikipedia with their field of activity and native language.

$R_{IT,2DRank}$	Person	Field	Culture
1	Pope John Paul II	Religion	WR
2	Pope Benedict XVI	Religion	DE
3	Giuseppe Garibaldi	Politics	IT
4	Raphael	Art	IT
5	Jesus	Religion	WR
6	Benito Mussolini	Politics	IT
7	Michelangelo	Art	IT
8	Leonardo da Vinci	Art	IT
9	Pier Paolo Pasolini	Art	IT
10	Michael Jackson	Art	EN
11	Martina Navratilova	Sport	EN
12	Saint Peter	Religion	WR
13	Pope Paul III	Religion	IT
14	Wolfgang Amadeus Mozart	Art	DE
15	John Lennon	Art	EN
16	Bob Dylan	Art	EN
17	Mina (singer)	Art	IT
18	William Shakespeare	Art	EN
19	Julius Caesar	Politics	IT
20	Titian	Art	IT
21	Silvio Berlusconi	Politics	IT
22	Alexander the Great	Politics	WR
23	Pablo Picasso	Art	ES
24	Antonio Vivaldi	Art	IT
25	Ludwig van Beethoven	Art	DE
26	Napoleon	Politics	FR
27	Madonna (entertainer)	Art	EN
28	Roger Federer	Sport	DE
29	Johann Sebastian Bach	Art	DE
30	Walt Disney	Art	EN

Table S12: Top 30 persons by CheiRank for Italian Wikipedia with their field of activity and native language.

$R_{IT,CheiRank}$	Person	Field	Culture
1	Ticone di Amato	Religion	WR
2	John the Merciful	Religion	WR
3	Nduccio	Art	IT
4	Vincenzo Olivieri	Art	IT
5	Leo Baeck	Religion	DE
6	Karl Wilhelm Reinmuth	Science	DE
7	Freimut Börngen	Science	DE
8	Nikolai Chernykh	Science	RU
9	Edward L. G. Bowell	Science	EN
10	Roger Federer	Sport	DE
11	Michel Morganella	Sport	WR
12	Rafael Nadal	Sport	ES
13	Robin Söderling	Sport	WR
14	Iván Zamorano	Sport	ES
15	Martina Navratilova	Sport	EN
16	Venus Williams	Sport	EN
17	Goran Ivanišević	Sport	WR
18	Javier Pastore	Sport	ES
19	Stevan Jovetić	Sport	WR
20	Mina (singer)	Art	IT
21	George Ade	Art	EN
22	Kazuro Watanabe	Sport	WR
23	Andy Roddick	Sport	EN
24	Johann Strauss II	Art	DE
25	Max Wolf	Science	DE
26	Isaac Asimov	Art	EN
27	Georges Simenon	Art	FR
28	Alice Joyce	Art	EN
29	Pietro De Sensi	Sport	IT
30	Noemi (singer)	Art	IT

Table S13: Top 30 persons by PageRank for Spanish Wikipedia with their field of activity and native language.

$R_{ES,PageRank}$	Person	Field	Culture
1	Carl Linnaeus	Scinece	WR
2	Napoleon	Politics	FR
3	Jesus	Religion	WR
4	Aristotle	Science	WR
5	Charles V, Holy Roman Emperor	Politics	ES
6	Adolf Hitler	Politics	DE
7	Julius Caesar	Politics	IT
8	Philip II of Spain	Politics	ES
9	William Shakespeare	Art	EN
10	Plato	Science	WR
11	Albert Einstein	Science	DE
12	Augustus	Politics	IT
13	Pope John Paul II	Religion	WR
14	Christopher Columbus	ETC	IT
15	Karl Marx	Science	DE
16	Alexander the Great	Politics	WR
17	Isaac Newton	Science	EN
18	Francisco Franco	Politics	ES
19	Charlemagne	Politics	FR
20	Immanuel Kant	Science	DE
21	Charles Darwin	Science	EN
22	Louis XIV of France	Politics	FR
23	Mary (mother of Jesus)	Religion	WR
24	Wolfgang Amadeus Mozart	Art	DE
25	Galileo Galilei	Science	IT
26	Cicero	Politics	IT
27	Homer	Art	WR
28	Paul the Apostle	Religion	WR
29	René Descartes	Science	FR
30	Miguel de Cervantes	Art	ES

Table S14: Top 30 persons by 2DRank for Spanish Wikipedia with their field of activity and native language.

$R_{ES,2DRank}$	Person	Field	Culture
1	Wolfgang Amadeus Mozart	Art	DE
2	Julius Caesar	Politics	IT
3	Simón Bolívar	Politics	ES
4	Francisco Goya	Art	ES
5	Madonna (entertainer)	Art	EN
6	Bob Dylan	Art	EN
7	Barack Obama	Politics	EN
8	Fidel Castro	Politics	ES
9	Michael Jackson	Art	EN
10	Richard Wagner	Art	DE
11	Augusto Pinochet	Politics	ES
12	Trajan	Politics	IT
13	Jorge Luis Borges	Art	ES
14	Juan Perón	Politics	ES
15	Porfirio Díaz	Politics	ES
16	Michelangelo	Art	IT
17	J. R. R. Tolkien	Art	EN
18	Paul McCartney	Art	EN
19	Adolf Hitler	Politics	DE
20	John Lennon	Art	EN
21	Hugo Chávez	Politics	ES
22	Elizabeth II	Politics	EN
23	Lope de Vega	Art	ES
24	Francisco Franco	Politics	ES
25	Christopher Columbus	ETC	IT
26	Diego Velázquez	Art	ES
27	Pablo Picasso	Art	ES
28	Edgar Allan Poe	Art	EN
29	Charlemagne	Politics	FR
30	Juan Carlos I of Spain	Politics	ES

Table S15: Top 30 persons by CheiRank for Spanish Wikipedia with their field of activity and native language.

$R_{ES,CheiRank}$	Person	Field	Culture
1	Max Wolf	Science	DE
2	Monica Bellucci	Art	IT
3	Che Guevara	Politics	ES
4	Steve Buscemi	Art	EN
5	Johann Palisa	Science	DE
6	Auguste Charlois	Science	FR
7	José Flávio Pessoa de Barros	Science	WR
8	Arturo Mercado	Art	ES
9	Francisco Goya	Art	ES
10	Bob Dylan	Art	EN
11	Jorge Luis Borges	Art	ES
12	Brian May	Art	EN
13	Virgilio Barco Vargas	Politics	ES
14	Mariano Bellver	ETC	ES
15	Demi Lovato	Art	EN
16	Joan Manuel Serrat	Art	ES
17	Mary Shelley	Art	EN
18	Ana Belén	Art	ES
19	Aki Misato	Art	WR
20	Carl Jung	Science	DE
21	Roger Federer	Sport	DE
22	Antoni Gaudí	Art	ES
23	Rafael Nadal	Sport	ES
24	Hans Melchior	Science	DE
25	Paulina Rubio	Art	ES
26	Paul McCartney	Art	EN
27	Julieta Venegas	Art	ES
28	Fermin Muguruza	Art	ES
29	Belinda (entertainer)	Art	ES
30	Patricia Acevedo	Art	ES

Table S16: Top 30 persons by PageRank for Dutch Wikipedia with their field of activity and native language.

$R_{NL,PageRank}$	Person	Field	Culture
1	Carl Linnaeus	Science	WR
2	Pierre Andre Latreille	Science	FR
3	Napoleon	Politics	FR
4	Eugene Simon	Science	FR
5	Jesus	Religion	WR
6	Charles Darwin	Science	EN
7	Julius Caesar	Politics	IT
8	Adolf Hitler	Politics	DE
9	Aristotle	Science	WR
10	Charlemagne	Politics	FR
11	Plato	Science	WR
12	Jean-Baptiste Lamarck	Science	FR
13	Ernst Mayr	Science	DE
14	Alexander the Great	Politics	WR
15	Louis XIV of France	Politics	FR
16	Pope John Paul II	Religion	WR
17	Alfred Russel Wallace	Science	EN
18	Charles V, Holy Roman Emperor	Politics	ES
19	Thomas Robert Malthus	Science	EN
20	Augustus	Politics	IT
21	William I of the Netherlands	Politics	NL
22	Joseph Stalin	Politics	RU
23	Albert Einstein	Science	DE
24	Beatrix of the Netherlands	Politics	NL
25	Christopher Columbus	Etc	IT
26	Elizabeth II	Politics	EN
27	Isaac Newton	Science	EN
28	Wolfgang Amadeus Mozart	Art	DE
29	J. B. S. Haldane	Science	EN
30	Cicero	Politics	IT

Table S17: Top 30 persons by 2DRank for Dutch Wikipedia with their field of activity and native language.

$R_{NL,2DRank}$	Person	Field	Culture
1	Pope Benedict XVI	Religion	DE
2	Elizabeth II	Politics	EN
3	Charles Darwin	Science	EN
4	Albert II of Belgium	Politics	NL
5	Albert Einstein	Science	DE
6	Pope John Paul II	Religion	WR
7	Michael Jackson	Art	EN
8	Johann Sebastian Bach	Art	DE
9	Saint Peter	Religion	WR
10	Johan Cruyff	Sport	NL
11	William Shakespeare	Art	EN
12	Christopher Columbus	Etc	IT
13	Augustus	Politics	IT
14	Frederick the Great	Politics	DE
15	Rembrandt	Art	NL
16	Eddy Merckx	Sport	NL
17	Ludwig van Beethoven	Art	DE
18	Pope Pius XII	Religion	IT
19	Peter Paul Rubens	Art	NL
20	Napoleon	Politics	FR
21	Wolfgang Amadeus Mozart	Art	DE
22	Igor Stravinsky	Art	RU
23	Martin of Tours	Religion	FR
24	Geert Wilders	Politics	NL
25	J.R.R. Tolkien	Art	EN
26	Pierre Cuypers	Art	NL
27	Charles V, Holy Roman Emperor	Politics	ES
28	Pope Pius IX	Religion	IT
29	Juliana of the Netherlands	Politics	NL
30	Elvis Presley	Art	EN

Table S18: Top 30 persons by CheiRank for Dutch Wikipedia with their field of activity and native language.

$R_{NL,CheiRank}$	Person	Field	Culture
1	Pier Luigi Bersani	Politics	IT
2	Francesco Rutelli	Politics	IT
3	Hans Renders	Science	NL
4	Julian Jenner	Sport	NL
5	Marten Toonder	Art	NL
6	Uwe Seeler	Sport	DE
7	Stefanie Sun	Art	WR
8	Roger Federer	Sport	DE
9	Theo Janssen	Sport	NL
10	Zazie	Art	FR
11	Albert II of Belgium	Politics	NL
12	Denny Landzaat	Sport	NL
13	Paul Biegel	Art	NL
14	Guido De Padt	Politics	NL
15	Jan Knippenberg	Sport	NL
16	Michael Schumacher	Sport	DE
17	Hans Werner Henze	Art	DE
18	Lionel Messi	Sport	ES
19	Johan Cruijff	Sport	NL
20	Eva Janssen (actrice)	Art	NL
21	Marion Zimmer Bradley	Art	EN
22	Graham Hill	Sport	EN
23	Rick Wakeman	Art	EN
24	Mihai Nesu	Sport	NL
25	Freddy De Chou	Politics	NL
26	Rubens Barrichello	Sport	WR
27	Ismail Aissati	Sport	NL
28	Marco van Basten	Sport	NL
29	Paul Geerts	Art	NL
30	Ibrahim Afellay	Sport	NL

Table S19: Top 30 persons by PageRank for Russian Wikipedia with their field of activity and native language.

$R_{RU,PageRank}$	Person	Field	Culture
1	Peter the Great	Politics	RU
2	Napoleon	Politics	FR
3	Carl Linnaeus	Science	WR
4	Joseph Stalin	Politics	RU
5	Alexander Pushkin	Art	RU
6	Vladimir Lenin	Politics	RU
7	Catherine the Great	Politics	RU
8	Jesus	Religion	WR
9	Aristotle	Science	WR
10	Vladimir Putin	Politics	RU
11	Julius Caesar	Politics	IT
12	Adolf Hitler	Politics	DE
13	Boris Yeltsin	Politics	RU
14	William Shakespeare	Art	EN
15	Ivan the Terrible	Politics	RU
16	Alexander II of Russia	Politics	RU
17	Nicholas II of Russia	Politics	RU
18	Karl Marx	Science	DE
19	Louis XIV of France	Politics	FR
20	Nicholas I of Russia	Politics	RU
21	Alexander I of Russia	Politics	RU
22	Alexander the Great	Politics	WR
23	Charlemagne	Politics	FR
24	William Herschel	Science	EN
25	Mikhail Gorbachev	Politics	RU
26	Paul I of Russia	Politics	RU
27	Leo Tolstoy	Art	RU
28	Nikolai Gogol	Art	RU
29	Dmitry Medvedev	Politics	RU
30	Lomonosov	Science	RU

Table S20: Top 30 persons by 2DRank for Russian Wikipedia with their field of activity and native language.

$R_{RU,2DRank}$	Person	Field	Culture
1	Dmitri Mendeleev	Science	RU
2	Peter the Great	Politics	RU
3	Justinian I	Politics	WR
4	Yaroslav the Wise	Politics	RU
5	Elvis Presley	Art	EN
6	Yuri Gagarin	Etc	RU
7	William Shakespeare	Art	EN
8	Albert Einstein	Science	DE
9	Adolf Hitler	Politics	DE
10	Christopher Columbus	Etc	IT
11	Catherine the Great	Politics	RU
12	Vladimir Vysotsky	Art	RU
13	Louis de Funes	Art	FR
14	Lomonosov	Science	RU
15	Alla Pugacheva	Art	RU
16	Viktor Yanukovych	Politics	RU
17	Nikolai Gogol	Art	RU
18	Felix Dzerzhinsky	Politics	RU
19	Aleksandr Solzhenitsyn	Art	RU
20	Pope Benedict XVI	Religion	DE
21	Maxim Gorky	Art	RU
22	Julius Caesar	Politics	IT
23	George Harrison	Art	EN
24	Bohdan Khmelnytsky	Politics	RU
25	Rembrandt	Art	NL
26	John Lennon	Art	EN
27	Jules Verne	Art	FR
28	Benito Mussolini	Politics	IT
29	Nicholas Roerich	Art	RU
30	Niels Bohr	Science	WR

Table S21: Top 30 persons by CheiRank for Russian Wikipedia with their field of activity and native language.

$R_{RU,CheiRank}$	Person	Field	Culture
1	Aleksander Vladimirovich Sotnik	Etc	RU
2	Aleksei Aleksandrovich Bobrinsky	Politics	RU
3	Boris Grebenshchikov	Art	RU
4	Karl Wilhelm Reinmuth	Science	DE
5	Ronnie O'Sullivan	Sport	EN
6	Max Wol	Science	DE
7	Ivan Egorovich Sizykh	Etc	RU
8	Vladimir Mikhilovich Popkov	Art	RU
9	Sun Myung Moon	Religion	KO
10	Mikhail Pavlovich Tolstoi	Etc	RU
11	Perry Como	Art	EN
12	John Heenan	Religion	EN
13	Petr Aleksandrovich Ivaschenko	Art	RU
14	Andrey Vlasov	Etc	RU
15	Christian Heinrich Friedrich Peters	Science	DE
16	Auguste Charlois	Science	FR
17	Damian (Marczhuk)	Religion	RU
18	Yuri Gagarin	Etc	RU
19	Stephen Hendry	Sport	EN
20	Ivan Grigorevich Donskikh	Etc	RU
21	Anna Semenovna Kamenkova-Pavlova	Art	RU
22	Ivan Nikolaevich Shulga	Art	RU
23	George Dwyer	Religion	EN
24	William Wheeler (bishop)	Religion	EN
25	Vladimir Vladimirovitsch Antonik	Art	RU
26	Leonid Parfyonov	Art	RU
27	Vincent Nichols	Religion	EN
28	Dmitri Mendeleev	Science	RU
29	Boris Vladimirovich Bakin	Etc	RU
30	George Harrison	Art	EN

Table S22: Top 30 persons by PageRank for Hungarian Wikipedia with their field of activity and native language.

$R_{HU,PageRank}$	Person	Field	Culture
1	Carl Linnaeus	Science	WR
2	Jesus	Religion	WR
3	Napoleon	Politics	FR
4	Aristotle	Science	WR
5	Julius Caesar	Politics	IT
6	Matthias Corvinus	Politics	HU
7	Szentagothai Janos	Science	HU
8	William Shakespeare	Art	EN
9	Adolf Hitler	Politics	DE
10	Stephen I of Hungary	Politics	HU
11	Augustus	Politics	IT
12	Michael Schumacher	Sport	DE
13	Miklos Rethelyi	Politics	HU
14	Sigismund, Holy Roman Emperor	Politics	HU
15	Lajos Kossuth	Politics	HU
16	Charles I of Hungary	Politics	HU
17	Bela IV of Hungary	Politics	HU
18	Maria Theresa	Politics	DE
19	Joseph Stalin	Politics	RU
20	Franz Joseph I of Austria	Politics	DE
21	Louis I of Hungary	Politics	HU
22	Francis II Rakoczi	Politics	HU
23	Mary (mother of Jesus)	Religion	WR
24	Sandor Petofi	Art	HU
25	Pope John Paul II	Religion	WR
26	Johann Wolfgang von Goethe	Art	DE
27	Alexander the Great	Politics	WR
28	Bela Bartok	Art	HU
29	Charlemagne	Politics	FR
30	Louis XIV of France	Politics	FR

Table S23: Top 30 persons by 2DRank for Hungarian Wikipedia with their field of activity and native language.

$R_{HU,2DRank}$	Person	Field	Culture
1	Stephen I of Hungary	Politics	HU
2	Sandor Petofi	Art	HU
3	Franz Liszt	Art	HU
4	Kati Kovacs	Art	HU
5	Alexander the Great	Politics	WR
6	Attila Jozsef	Art	HU
7	Aristotle	Science	WR
8	Kimi Raikkonen	Sport	WR
9	Rubens Barrichello	Sport	WR
10	Lajos Kossuth	Politics	HU
11	Bela Bartok	Art	HU
12	Charlemagne	Politics	FR
13	Sandor Weores	Art	HU
14	Mariah Carey	Art	EN
15	Wolfgang Amadeus Mozart	Art	DE
16	Josip Broz Tito	Politics	WR
17	Charles I of Hungary	Politics	HU
18	Isaac Asimov	Art	EN
19	Napoleon	Politics	FR
20	Bonnie Tyler	Art	EN
21	Miklos Radnoti	Art	HU
22	Jay Chou	Art	WR
23	Janos Kodolanyi	Art	HU
24	Louis I of Hungary	Politics	HU
25	Zsuzsa Koncz	Art	HU
26	Adolf Hitler	Politics	HU
27	Stephen King	Art	EN
28	Mor Jokai	Art	HU
29	Ferenc Erkel	Art	HU
30	Franz Joseph I of Austria	Politics	DE

Table S24: Top 30 persons by CheiRank for Hungarian Wikipedia with their field of activity and native language.

$R_{HU,CheiRank}$	Person	Field	Culture
1	Edward L. G. Bowell	Science	EN
2	Karl Wilhelm Reinmuth	Science	DE
3	Max Wolf	Science	DE
4	Benjamin Boukpeti	Sport	FR
5	Urata Takesi	Science	WR
6	Wilfred Bungei	Sport	WR
7	Henri Debehogne	Science	FR
8	Lee "Scratch" Perry	Art	WR
9	Karl Golsdorf	Etc	DE
10	Johann Palisa	Science	DE
11	Dirk Kuijt	Sport	NL
12	Roger Federer	Sport	DE
13	Csernus Imre	Etc	HU
14	Kati Kovacs	Art	HU
15	Rafael Nadal	Sport	ES
16	Venus Williams	Sport	EN
17	Sebastien Loeb	Sport	FR
18	Pleh Csaba	Science	HU
19	Tibor Antalpeter	Sport	HU
20	Serena Williams	Sport	EN
21	Csore Gabor	Art	HU
22	Pirmin Schwegler	Sport	DE
23	Olivia Newton-John	Art	EN
24	Petter Solberg	Sport	WR
25	Orosz Anna	Art	HU
26	Zsambeki Gabor	Art	HU
27	Vera Igorevna Zvonarjova	Sport	RU
28	Sandor Petofi	Art	HU
29	Roberta Vinci	Sport	IT
30	Flavia Pennetta	Sport	HU

Table S25: Top 30 persons by PageRank for Korean Wikipedia with their field of activity and native language.

$R_{KO,PageRank}$	Person	Field	Culture
1	Carl Linnaeus	Science	WR
2	Gojong of the Korean Empire	Politics	KO
3	Jesus	Religion	WR
4	John Edward Gray	Science	EN
5	Aristotle	Science	WR
6	Napoleon	Politics	FR
7	Sejong the Great	Politics	KO
8	Park Chung-hee	Politics	KO
9	Emperor Wu of Han	Politics	WR
10	Seonjo of Joseon	Politics	KO
11	Taejong of Joseon	Politics	KO
12	Syngman Rhee	Politics	KO
13	Kim Dae-jung	Politics	KO
14	Roh Moo-hyun	Politics	KO
15	Yeongjo of Joseon	Politics	KO
16	Adolf Hitler	Politics	DE
17	Taejo of Joseon	Politics	KO
18	Sukjong of Joseon	Politics	KO
19	Kim Il-sung	Politics	KO
20	Qianlong Emperor	Politics	WR
21	Kim Jong-il	Politics	KO
22	Kangxi Emperor	Politics	WR
23	Emperor Gaozu of Han	Politics	WR
24	Chun Doo-hwan	Politics	KO
25	Taejo of Goryeo	Politics	KO
26	George W. Bush	Politics	EN
27	Qin Shi Huang	Politics	WR
28	Jeongjo of Joseon	Politics	KO
29	Sunjo of Joseon	Politics	KO
30	Cao Cao	Politics	WR

Table S26: Top 30 persons by 2DRank for Korean Wikipedia with their field of activity and native language.

$R_{KO,2DRank}$	Person	Field	Culture
1	Gojong of the Korean Empire	Politics	KO
2	Sejong the Great	Politics	KO
3	Park Chung-hee	Politics	KO
4	Taejong of Joseon	Politics	KO
5	Kim Dae-jung	Politics	KO
6	Roh Moo-hyun	Politics	KO
7	Syngman Rhee	Politics	KO
8	Kim Il-sung	Politics	KO
9	Qianlong Emperor	Politics	WR
10	Kangxi Emperor	Politics	WR
11	Taejo of Goryeo	Politics	KO
12	Seonjo of Joseon	Politics	KO
13	Jeongjo of Joseon	Politics	KO
14	Kim Young-sam	Politics	KO
15	Julius Caesar	Politics	IT
16	Chun Doo-hwan	Politics	KO
17	Injo of Joseon	Politics	KO
18	Tokugawa Ieyasu	Politics	WR
19	Lee Myung-bak	Politics	KO
20	Seongjong of Joseon	Politics	KO
21	Cao Cao	Politics	WR
22	Confucius	Science	WR
23	Mao Zedong	Politics	WR
24	Taejo of Joseon	Politics	KO
25	Toyotomi Hideyoshi	Politics	WR
26	Heungseon Daewongun	Politics	KO
27	Liu Bei	Politics	WR
28	Yeongjo of Joseon	Politics	KO
29	Pope John Paul II	Religion	WR
30	Adolf Hitler	Politics	DE

Table S27: Top 30 persons by CheiRank for Korean Wikipedia with their field of activity and native language.

$R_{KO,CheiRank}$	Person	Field	Culture
1	Lee Jong-wook (baseball)	Sport	KO
2	Kim Dae-jung	Politics	KO
3	Lionel Messi	Sport	ES
4	Kim Kyu-sik	Politics	KO
5	Johannes Kepler	Science	DE
6	Yun Chi-young	Politics	KO
7	Michael Jackson	Art	EN
8	Yi Sun-sin	ETC	KO
9	Chang Myon	Politics	KO
10	IU (singer)	Art	KO
11	Kim Seo-yeong	Art	KO
12	Tokugawa Ieyasu	Politics	WR
13	Jeremy Renner	Art	EN
14	Zhao Deyin	Politics	WR
15	Yang Joon-Hyu	Sport	KO
16	Zhang Gui (Tang Dynasty)	Politics	WR
17	Zinedine Zidane	Sport	FR
18	Park Chung-hee	Politics	KO
19	Heungseon Daewongun	Politics	KO
20	Ahn Ji-hwan	Art	KO
21	Lee Seung-Yeop	Sport	KO
22	Roh Moo-hyun	Politics	KO
23	Britney Spears	Art	EN
24	Kim Young-sam	Politics	KO
25	Jeong Hyeong-don	Art	KO
26	Kim Yu-Na	Sport	KO
27	Park Jong-Seol	Art	KO
28	Lim Taekyoung	Art	KO
29	Park Ji-Sung	Sport	KO
30	Yuh Woon-Hyung	Politics	KO
